# Neuroimaging correlates of suicidal behavior in dementia

**DOI:** 10.1192/j.eurpsy.2021.2191

**Published:** 2021-08-13

**Authors:** I. Mudrenko, O. Kolenko

**Affiliations:** Department Of Neurosurgery And Neurology, Sumy State University, Sumy, Ukraine

**Keywords:** suicidal behavior, neuroimaging changes, dementia.

## Abstract

**Introduction:**

At pathomorphological research of suicides reveal neurogenerative changes, which determines the relevance of the search for neuroimaging predictors (suicidal behavior) SB in dementia.

**Objectives:**

To study predictors of SB (in dementia, due to Alzheimer’s disease (AD), vascular (VD), mixed (MD) based on neuroimaging research.

**Methods:**

We examined 213 patients with dementia in АD, VD, MD on a CT, which were divided by the factor of the presence of SB into the main and control groups.

**Results:**

At patients with SB at AD expansion of basal cistern is revealed of the brain (59%), but signs of chronic ischemia in the form of a decrease in the density of brain matter in the projection of the basal ganglia and white matter (67%), dilation of the ventricular system of the brain (51%), more typical for patients without SB. At VD with SB periventricular leukoareosis (67%), expansion of subarachnoid spaces (82%) and deepening of cracks of a brain (67%) are found. Patients without SB were characterized by a decrease in the density of brain matter in the projection of the white matter (73%). At MD with SB expansion of subarachnoid spaces (100%), basal cistern of a brain (87%), periventricular leukoareosis (87%), decrease in density of substance in the brain were registered in the projection of the basal ganglia (100%). Patients without SB with MD had deepening of the brain slits (40%).

**Conclusions:**

Neuroimaging signs in the form of chronic ischemia and pronounced atrophic changes in the brain are factors in the anti-risk of SB in dementia.

**Disclosure:**

No significant relationships.

